# Associations of cognitive impairment in patients with schizophrenia with genetic features and with schizophrenia-related structural and functional brain changes

**DOI:** 10.3389/fgene.2022.880027

**Published:** 2022-08-19

**Authors:** Chuanjun Zhuo, Hongjun Tian, Jiayue Chen, Qianchen Li, Lei Yang, Qiuyu Zhang, Guangdong Chen, Langlang Cheng, Chunhua Zhou, Xueqin Song

**Affiliations:** ^1^ Key Laboratory of Real Time Tracing of Brian Circuits in Psychiatry and Neurology (RTBNP_Lab), Nankai University Affiliated Tianjin Fourth Center Hospital, Tianjin Fourth Center Hospital, Tianjin, China; ^2^ Digital Analysis Center of Psychiatry, Tianjin Fourth Center Hospital, Tianjin, China; ^3^ Department of Psychiatry and Neurology Imaging-Genetics and Comorbidity Laboratory (PNGC_Lab) of Tianjin Mental Health Center, Tianjin Anding Hospital, Tianjin, China; ^4^ Department of Psychiatry, The First Affiliated Hospital of Zhengzhou University, Zhengzhou, China; ^5^ Deep Learning Center of MRI and Genetics, Wenzhou Seventh People’s Hospital, Wenzhou, China; ^6^ Department of Psychiatry, Nankai University Affiliated Tianjin Fourth Center Hospital, Tianjin Fourth Center Hospital, Tianjin, China; ^7^ Department of Pharmacology, The First Hospital of Hebei Medical University, Shijiazhuang, China; ^8^ Department of Psychiatry, Wenzhou Seventh People’s Hospital, Wenzhou, China

**Keywords:** schizophrenia, SNP, cognitive impairment, GMV, FC

## Abstract

Cognitive impairment is highly prevalent in patients with major psychiatric disorders (MPDs), including schizophrenia (SCZ), bipolar disorder, major depressive disorder, in whom it can be highly disruptive to community functioning and worsen prognosis. Previously, genetic factors and cognitive impairments in MPD patients have been examined mostly in isolated circuits rather than in the whole brain. In the present study, genetic, neuroimaging, and psychometric approaches were combined to investigate the relationship among genetic factors, alterations throughout the brain, and cognitive impairments in a large cohort of patients diagnosed with SCZ, with a reference healthy control (HC) group. Single nucleotide polymorphisms (SNPs) in SCZ-risk genes were found to be strongly related to cognitive impairments as well as to gray matter volume (GMV) and functional connectivity (FC) alterations in the SCZ group. Annotating 136 high-ranking SNPs revealed 65 affected genes (including *PPP1R16B, GBBR2*, *PDE4B*, *CANCNA1C*, *SLC12AB*, *SATB2*, *MAG12*, and *SATB2*). Only one, a *PDE4B* SNP (rs1006737), correlated with GMV (*r* = 0:19 *p* = 0.015) and FC (*r* = 0.21, *p* = 0.0074) in SCZ patients. GMV and FC alterations correlated with one another broadly across brain regions. Moreover, the present data demonstrate three-way SNP-FC-GMV associations in patients with SCZ, thus providing clues regarding potential genetic bases of cognition impairments in SCZ. SNP-FC-GMV relationships correlated with visual learning and reasoning dimensions of cognition. These data provide evidence that SCZ-related cognitive impairments may reflect genetically underlain whole-brain structural and functional alterations.

## Introduction

Schizophrenia (SCZ), which has a worldwide prevalence of about 1%, is a highly debilitating major psychiatric disorder (MPD) that, in addition to psychosis symptoms, commonly involves impaired cognition (Insel, 2010). Cognition refers to the diverse assembly of abilities that allow an individual to recognize, process, and respond to information (Smeland et al., 2020). Consequently, cognitive impairment alters how one perceives, processes, and reacts to external information, which can lead to bizarre thinking, soundtrack language, and abnormal behavior (González-Blanch et al., 2006). Among patients with MPDs, impaired cognition is seen most prominently in patients with SCZ (60–80%) (González-Blanch et al., 2006; Smeland et al., 2020) though it is also observed in patients with bipolar disorder (Jenkins, 2019; Zhu et al., 2019) and depression (Miskowiak et al., 2016).

Cognitive ability is an important consideration in patients with MPDs because it affects their ability to function within the community (Reilly and Sweeney, 2014). Cognitive impairment has been observed by several groups to precede MPD onset and then to deteriorate within the first 2–3 years that patients are medicated, causing challenges even in remission (Allen et al., 2003; Ivleva et al., 2010; McCleery and Nuechterlein, 2019). The impairment severity appears to stabilize after 5–10 years of treatment (Holthausen et al., 2002; Allen et al., 2003; Keefe et al., 2005; Vaskinn et al., 2014). Cognitive impairment in patients with SCZ specifically has been reported to be accompanied by a 2% loss in gray matter (GM) and a 1% loss in white matter (WM), accounting for a whole-brain volumetric loss of about 2.6% within 2 years of illness onset, followed by relative stability (Vita et al., 2012). SCZ-associated cognitive impairments have been demonstrated in the important realms of attention, memory, language, executive function, and social cognitive function (Nuechterlein et al., 2004), and GM alterations have been related to cognitive impairment and brain functional alterations in schizophrenic patients with cognitive impairments (Jauhar et al., 2022).

There has been a diversity of evidence regarding potential mechanisms of cognitive impairment in MPDs, especially SCZ. For example, hippocampus-prefrontal lobe linking theta oscillations have been shown to be related to cognitive performance in patients with SCZ (Soltani Zangbar et al., 2020). Additionally, the functional magnetic resonance imaging (fMRI) findings in patients with SCZ reported by Luck and Vogel, (2013) revealed hypoactivity in memory-related brain regions and hyperactivity in other brain regions. Zaytseva et al. (2018) related SCZ-related cognitive impairments to localized aberrations in brain activity that lead gradually to the disturbance of whole-brain networks (Zaytseva et al., 2018; Luck et al., 2019). Sutcliffe et al. (2016) suggested that the cognitive impairments may reflect an interaction of risk genes with activity disturbances in the anterior cingulate and dorsolateral prefrontal cortex. These various lines of evidence have in common an indication that cognitive impairments in SCZ reflect a whole-brain systemic disturbance. Hence, elucidating the underlying pathological features should be based on whole-brain systemic theory.

Cognitive impairments in patients with SCZ have been linked to genetic single nucleotide polymorphisms (SNPs) (Eum et al., 2021). For example, in a meta-analysis, Rees et al. (2014) identified five SNPs as risk factors for SCZ-associated cognitive impairment: 1q21.1del; 1q21.1dupl; NRXN1 del; 16p11.2 dup; and 22q11.2 del. Waddington et al. (2021) reported that expression of dysbindin, encoded by the SCZ-risk associated gene *DTNBP1* (dystrobrevin-binding protein-1) may be related to cognitive impairments in SCZ, especially impairments affecting working memory, attention, and executive function. In addition, Matosin et al. (2018) reported evidence relating cognitive impairment to aberrant regulation of glutamate metabotropic receptor subtype 5, encoded by *GRM5*; and Iwamoto and Ouchi (2014) reported that insulin-like growth factor 2 can influence cognitive performance. In their meta-analysis, Bozza et al. (2013) found that loss of the 6p25 locus can induce serious cognitive impairments, especially in speech and language. The authors of these above-mentioned studies all discussed cognitive impairment preceding SCZ onset; furthermore, some even noted cognitive impairments in non-schizophrenic individuals with the identified SNPs who were at high risk of SCZ (i.e., those with a first degree relative with SCZ).

In the last two decades, there has been growth in research focused on investigating cognition impaired in patients with SCZ, most notably including recent studies employing genome-wide association study (GWAS) and neuroimaging techniques and relating detected gene variants and brain alterations to cognitive performance. For example, Bhattacharyya et al. found that 12 SNPs in human accelerated regions were associated with cognitive performance in the patients with SCZ (Maria Teixeira et al., 2020). Chen et al. reported evidence suggesting that the rs3811655 variant allele of *TF* may act as a moderator of the association between Cu/Zn-SOD activity and cognition (Mancuso et al., 2021), whereas Zakowicz et al. (2022). Reported that the rs1800871 and rs18008729 variant alleles of *IL10* were highly predictive of poor cognitive performance in patients with SCZ (Smeland et al., 2017). Fu et al. (2021) reported that the *CMIP* allele variant rs2287112 was related to cognition ability in patients with SCZ. Based on GWAS data, Smeland et al. (2017) who have related numerous genetic loci to cognitive traits, including intelligence, general cognition, reaction time, and verbal-numerical reasoning identified 21 loci that influence both SCZ and cognitive traits, including 2 loci that influence verbal-numerical reasoning, 6 loci that influence reaction time, and 14 that influence general cognitive function. One particularly relevant SCZ-associated locus was related to two cognitive traits (Jenkins, 2019). Studies using advanced neuroimaging techniques have revealed structural and functional alterations in the brain that can be related to SNPs and cognitive deficits. For example, Dixon et al. reported that decreased GABA or glutamate concentrations in frontal lobe areas correlated with cognitive performance in patients with SCZ (Miskowiak et al., 2016), Picó-Pérez et al. reported that structural aberrations in the neocortex (frontal, parietal, occipital, and temporal lobes) and in subcortical structures (amygdala, nucleus accumbens, caudate, putamen, and pallidum) correlated with cognitive dysfunction in patients with SCZ (Reilly and Sweeney, 2014). Dienel et al. (2022) found that genetic changes associated with altered excitatory signaling in layer 3 pyramidal neurons were associated with cognitive impairments in the prodromal phase of SCZ (Ivleva et al., 2010). Importantly, Mahmoudi et al. (2021) reported that rs58335419, a variable number tandem repeat variant of *MIR137*, is associated with cognitive impairment and altered cortical morphology in patients with SCZ (McCleery and Nuechterlein, 2019). Schweiger et al. (2019) reported that rs6265, a missense *BDNF* SNP allele, was associated with impaired cognition in the patients with SCZ (Allen et al., 2003). Yu et al. (2016) reported that the rs1063843 allele of *CAMKK2* was disruptive to dorsolateral prefrontal cortex functional activity and associated with reduced cognitive ability in the patients with SCZ. Future neuroimaging studies may help to clarify the aforementioned and other yet to be identified genetic alternations affect the brains of patients with SCZ.

The aim of this study was to use genetic, neuroimaging, and psychometric techniques to explore how genetics may relate to brain alterations and cognitive impairments in patients with SCZ. Brain alterations were assessed in terms of differences in gray matter volume (GMV) and functional connectivity (FC). We hypothesized that: 1) genetic factors can be associated with SCZ-related cognitive impairment; 2) brain structural and functional alterations, represented as GMVs and FCs, can be associated with cognitive impairments; and 3) genetic factors and brain alterations may interact in causing cognitive impairments in patients with SCZ.

## Materials and methods

### Subjects

We recruited 80 healthy control (HC) subjects and 78 patients with schizophrenia (SCZ) to participate in this study. All participants were recruited from the Psychiatric Department at Tianjin Fourth Centre Between 1 March 2020 and 28 February 2021. The Ethics committee of Tianjin Fourth Center Hospital approved this study. All participants signed informed consent forms.

The inclusion criteria for the SCZ group were as follows: confirmed diagnosis by two psychiatrists using the SCID (Structured Clinical Interview for DSM Disorders) according to the criteria of DSM-IV (Diagnostic and Statistical Manual of Mental Disorders, Fourth Edition) criteria; 18–35 years old; no diagnostic history of any neurological comorbidity or serious other comorbidity, including diabetes; no history of drug or alcohol dependence; and no contraindications for magnetic resonance scanning.

The HC group participants were matched with SCZ case participants with respect to age and gender, had no history of neurological or mental illness, and no family history of MPD within three generations of immediate relatives (screened with the SCID non-patient edition).

Additionally, to remove the potential influence of abnormal blood glucose on cognitive performance in this study (28, 29), we excluded potential participants meeting the following definition of prediabetes: >5.6 mmol/L *or* plasma glucose of 2 hours after meal >7.8 mmol/L. After excluding participants meeting either component of this definition (i.e. 45 SCZ patients and 46 HCs), we included 33 SCZ patients and 34 HCs in the final analysis.

### Genetic data acquisition

All participants provided blood samples for genetic analysis. Genomic DNA was extracted via the standard protocol (protease K digestion, phenol-chloroform extraction, and ethanol precipitation) and submitted to whole-genome genotyping of 571,054 loci with Infinium™ PsychArray-24 v1.3 BeadChip kits (Illumina China).

### Imaging data acquisition

Structural data and functional data were acquired with a 3.0-T Siemens trio scanner at the Medical Imaging Department of Tianjin Fourth Central Hospital. Soft foam pads were fitted to each subject’s head to reduce head movement and scanner noise. All participants were asked to keep their eyes closed and to relax during scanning, but do not fall asleep, and to avoid head movements as much as possible. The scanning parameters for resting fMRI were: repetition time (TR), 2000 ms; echo time (TE), 30 ms; flip angel, 90°; matrix, 64 × 64; field of view (FOV), 220 × 220; slice thickness, 4 cm; and voxel size 3.4 × 3.4 × 3.4. The scanning parameters for structural magnetic resonance image were: TR, 2530 ms; TE, 3.4 ms; flip angel, 7°; matrix, 256 × 256; FOV, 256 × 256; slice thickness, 1.0 cm; and voxel size 1 × 1 × 1 (Zhuo et al., 2017).

### Imaging data preprocessing

Structural data were analyzed by voxel-based morphology in SPM8 software (www.fil.Ion.UCL. Ac.uk/SPM)/Software/spm). Voxel-based morphology data preprocessing included the following steps: 1) three-dimensional images were registered in Montreal Neurological Institute standard space; 2) brain tissue within the images was divided into GM, WM, and cerebrospinal fluid (CSF) sections according to *a priori* probability and image gray information; 3) spatial registration-related brain volume alterations were corrected by modulation; 4) Gaussian smoothing was applied to suppress the signal-to-noise ratio. Subsequently, to adapt to the correlation analysis algorithm, subject’s GMV voxel values were aligned in a row vector to obtain a characteristic matrix integrated by the row vectors of all subjects within a group (Zhuo et al., 2017).

### GMV determination

GMVs were determined in SPM8 software (http://www.fil.ion.ucl.ac.uk/spm/software/spm8/). In brief, the standard unified segmentation model was used for segmentation of structural MR images into GM, WM, and CSF. Following an initial affine registration of the GM concentration map into the MNI space, diffeomorphic anatomical registration with the exponentiated lie algebra technique was used to warp GM concentration images nonlinearly, and then the imaging data were resampled at a cubic voxel size of 3 mm^3^. GMVs were calculated voxel-wise by multiplying the GM concentration map by the non-linear determinants from the spatial normalization step. GMV images were smoothed with a full-width at half maximum cubic Gaussian kernel (6 mm^3^) and subjected to spatial preprocessing to obtain smoothed GM maps for statistical analyses (Zhuo et al., 2017).

### FC analysis

FC analyses were conducted in SPM8 (http://www.fil.ion.ucl.ac.uk/spm/software/spm8/). Briefly, the first ten volumes were eliminated. The functional images were slice-time corrected, realigned, normalized to a standard MNI space, and re-sliced to a 3-mm slice thickness. Twenty-four motion parameters, together with linear drift, CSF, and WM signals, were then regressed out, filtered (0.01–0.08 Hz), and smoothed with a full-width half of maximum of 6 mm × 6 mm × 6 mm. We defined 256 FC matrix elements and binary FC matrix elements based on the 246 Brainnetome regions, including subcortical nuclei. The 1% most-positive FCs were retained and then their associated network properties were examined with the Brain Connectivity Toolbox as described by Meunier et al. (2020). We calculated FC modularity according to the binary FC matrix (Tohka et al., 2016) to ensure consistency across groups. Subsequently, the binary FC matrix then corresponded to eight Yeo networks based on module segregation index values by Hsu et al. (2020) method. Intranetwork and internetwork connections in and among the eight Yeo networks were quantitated as described in detail in our recent work (Xuan et al., 2022).

### Global functional connectivity density calculation

We calculated a gFCD value for each voxel using an in-house Linux script. FCs were compared across voxels with Pearson’s linear correlation analysis (correlation coefficient threshold, R > 0.6). On the basis of the whole-brain GMV, the gFCD of each voxel (×0) was calculated with a growth algorithm in accordance with the total FC [k (x0)] between x0 and all other voxels. To improve the normality of the gFCD value distribution, each gFCD value was divided by the mean gFCD of all included voxels. During gFCD determination, FCD maps were smoothed with a 6 mm × 6 mm × 6 mm Gaussian kernel to minimize functional anatomy differences as described previously (Yao et al., 2021).

### Association among GMV, FC, and gFCD calculation

Combination analysis was employed to confirm the predominance of SNP-GM-FC correlation and the existence of group differences and to test whether other factors, such as group information, do interfere with inter-modal correlation. Pair-wise inter-modal correlations were determined after controlling for age, gender, education level, and group (Li et al., 2021).

### Data analysis

Inter-group variances in MCCB scores and GMVs were detected with analyses of variance (ANOVAs). Inter-group differences in FC and gFCD were detected with student test. Pearson correlational analyses were conducted across variables. Statistical tests were conducted in SPSS version 21.0 (IBM, United States).

## Results

### Cognitive impairments

ANOVAs demonstrated that group (SCZ vs. HC) had a main effect on all eight MCCB dimension subscores and the MCCB composite score (Table 1).

**TABLE 1:** Reporting and comparison of demographics, clinical characteristics, and psychometric variables across schizophrenia (SCZ) and healthy control (HC) groups.

Variable	HC (N = 34)	SCZ (N = 33)	*P*
*Demographics*			
Gender, no. male/female	16/18	13/20	0.014
Age, years	25.9 ± 2.8	26.1 ± 3.5	0.231
Education level, years	12.6 ± 4.2	16.8 ± 5.5	0.119
*Clinical characteristics (SCZ only)*			
Duration of illness, years	–	5.6 ± 0.8	–
Medication dosage, mg/day^a^	–	655.0 ± 90.8	–
*Psychometrics*			
PANSS scores	–	93.2 ± 6.9	–
MCCB scores			
Speed procession	46.25 ± 4.25	38.23 ± 1.22	0.013
Attention vigilance	45.36 ± 6.23	35.36 ± 6.03	0.042
Working memory	45.55 ± 6.02	36.44 ± 2.15	0.015
Verbal learning	42.22 ± 3.26	36.33 ± 2.00	0.017
Visual learning	44.59 ± 3.94	32.55 ± 2.45	0.001
Reasoning	45.00 ± 3.00	36.54 ± 2.01	0.012
Social recognition	42.55 ± 5.84	34.15 ± 0.99	0.014
Composite	47.22 ± 3.59	32.43 ± 2.05	0.003

a Medication dosage is reported as the chlorpromazine equivalent dosage.

### GMV alterations

ANOVAs showed that, compared to the HC group, the SCZ group had significantly decreased GMVs in the hippocampus, temporal lobe, amygdala, and cerebellum.

### FC and gFCD alterations

**FIGURE 1:**
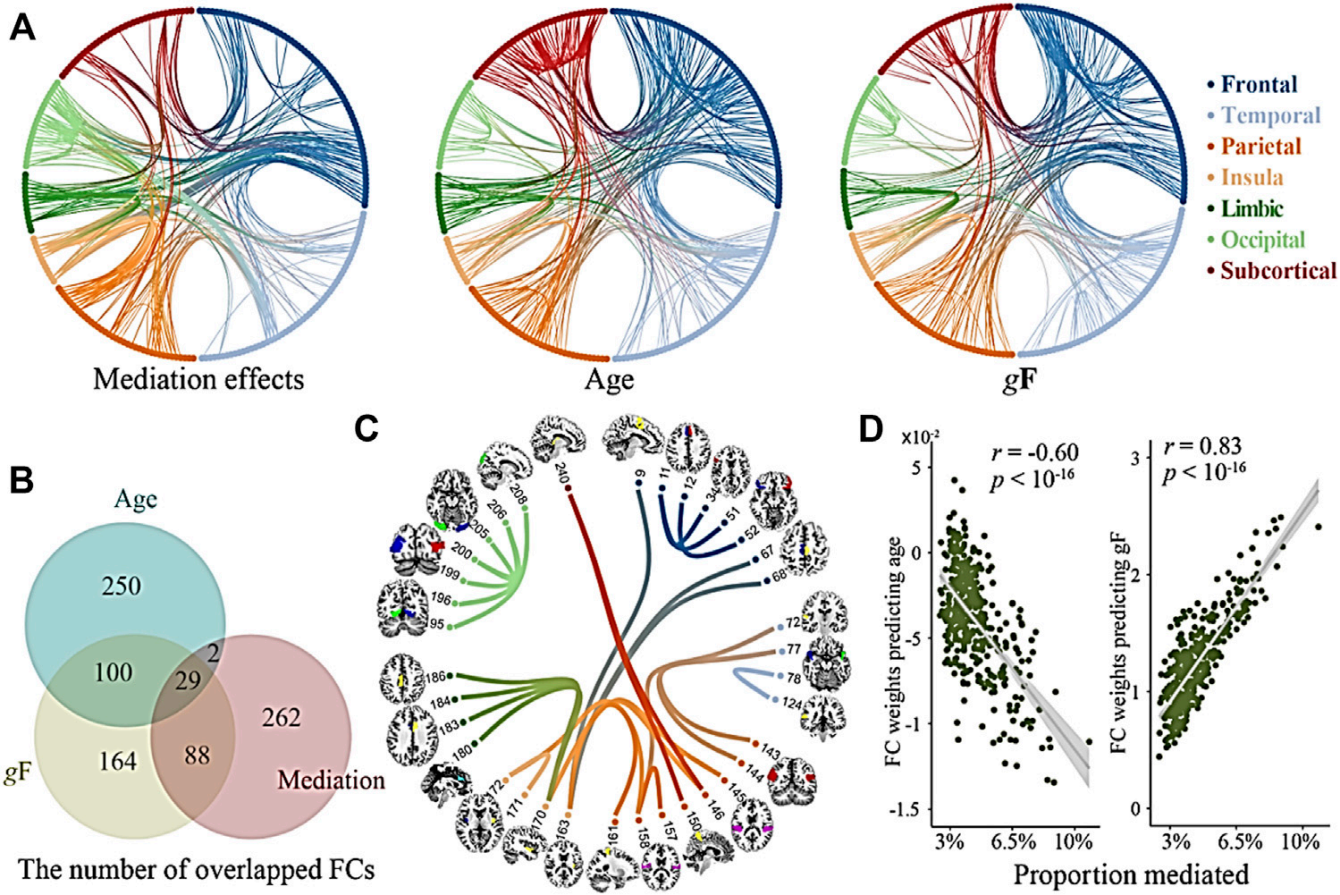
Coupling of GMV and gFCD alterations. **(A)** The mediations effects of GMV and gFCD when age and global structural connection (gF) are regressed out. **(B)** The numbers of overlapped GMV and gFCD connections (age, mediation effects, and gF are regress out). **(C)** Overlapped GMV and gFCD are associated with cognitive deficits. **(D)** Proportion mediated of age and global structural connections (gF) in the correlations of total functional connectivity (FC).

**FIGURE 2:**
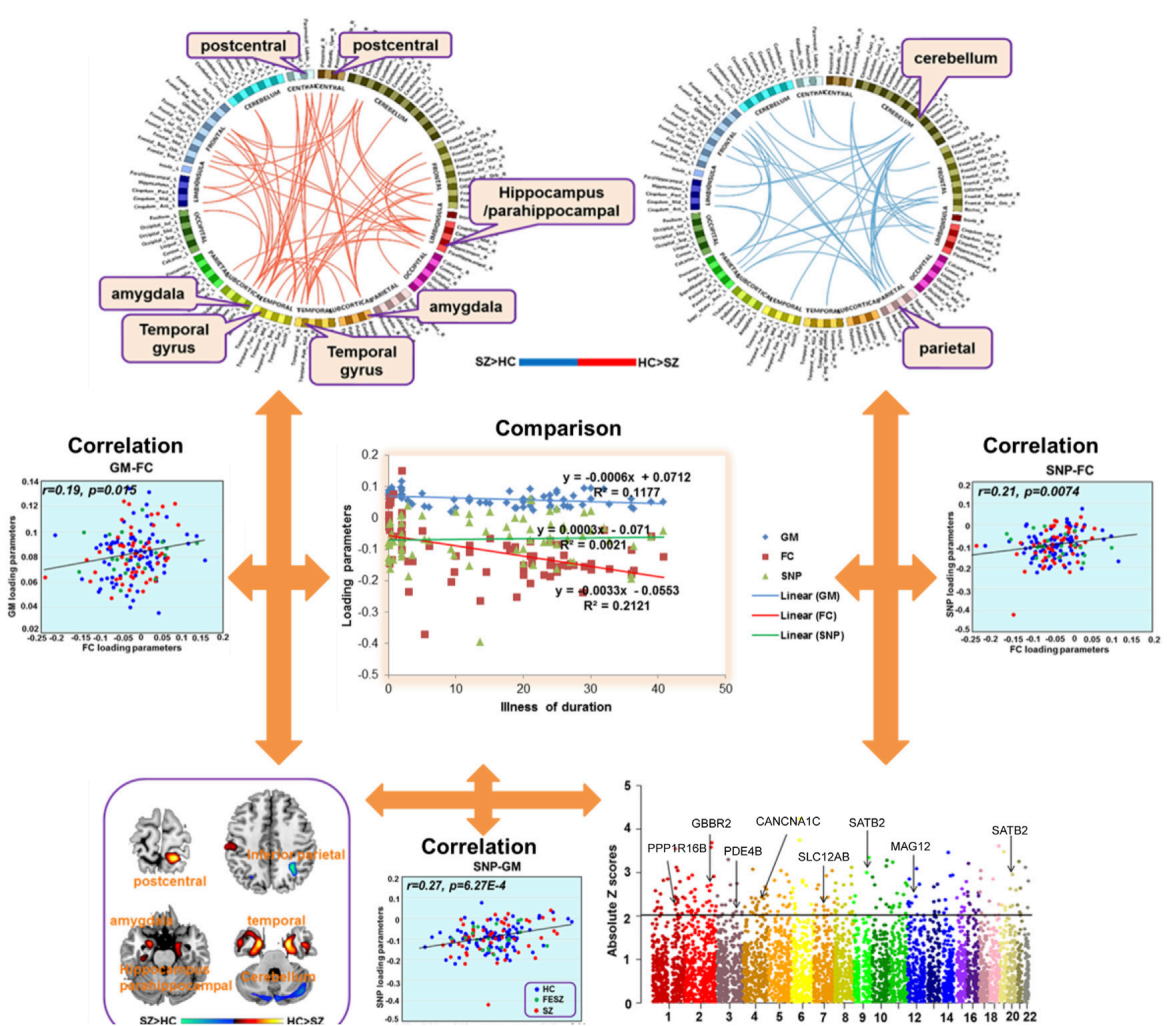
Relationships of SNP to GMV and FC alterations throughout the brain.

**FIGURE 3:**
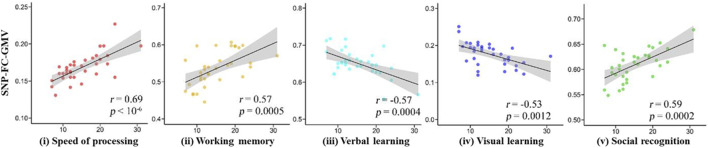
Five regions of interest with strong gFCD correlations with MCCB scores.

Compared to the HC group, the SCZ group had decreased FC between the postcentral gyrus and amygdala, increased FC between the parietal lobe and cerebellum (Figure 1), increased gFCD in the orbital frontal lobe, medial prefrontal cortex, parietal lobe, and right temporal lobe, and decreased gFCD in the left temporal lobe and bilateral hippocampus. Follow-up region-of-interest analyses of brain regions with aberrant gFCD in the SCZ group demonstrated that basal ganglia FC with the postcentral gyrus and left temporal lobe was decreased in the SCZ group while basal ganglia FC with the parietal lobe and occipital lobe was increased in the SCZ group (Figure 2), with the latter increases correlating significantly with PANSS negative symptoms scores in the SCZ group (Figure 3).

### Genetic alterations

The genetic data were preprocessed in PLINK, applying quality control procedures, and overlapped with PGC’s SCZ2 database. Applying a significance threshold of *p* < 0.01 yielded 4,937 SNPs. Discrete numbers were assigned to the categorical genotypes: 0, no minor allele; 1, one minor allele; and 2, two minor alleles. SNP components were transformed to Z scores and visualized at |Z| > 2. Applying this cutoff yielded 258 SNPs, of which 136 were high-ranking, involving 101 genes, including *SATB2*, *GABBR2*, *PDE4B*, and *CACNA1C*, among others (Table 2; Figure 2).

**TABLE 2:** High-ranking SNPs with |Z| > 2 (*n* = 136).

Chr	SNP	BP	MA	Gene	Z	Chr	SNP	BP	MA	Gene	Z
1	rs4916014	65421058	A	*JAK1*	−2.06	8	rs10093390	26142995	A		2.27
1	rs1419305	223912748	A	*CAPN2*	2.06	8	rs7004035	80674788	A		2.27
1	rs6658382	192605325	G	*RGS13*	−2.05	8	rs7011502	60705885	G		−2.21
2	rs7534143	66470379	C	*PDE4B*	4.15	8	rs2979164	8291357	A		−2.76
2	rs2372957	217927057	A		−2.92	8	rs2113664	113761143	A	*CSMD3*	−2.08
2	rs878292	29258193	A	*FAM179A*	−2.89	8	rs11773913	10470915	G	*RP1L1*	2.07
2	rs1867945	29277200	G		2.86	8	rs10505549	131278618	A	*ASAP1*	−2.06
2	rs895526	200162425	A	*SATB2*	2.72	9	rs10817792	118311318	A		−3.34
2	rs1518548	50932942	G	*NRXN1*	−2.55	9	rs4741148	11339109	A		−2.02
2	rs10204821	42027041	A		−2.53	10	rs1889567	112266771	A	*DUSP5*	−3.28
2	rs6546796	73247148	A	*SFXN5*	−2.43	10	rs7087113	106202531	A	*CCDC147*	−3.15
2	rs811526	79227099	A		2.34	10	rs4745849	64313875	C	*ZNF365*	−2.38
2	rs7599152	167759948	G	*XIRP2*	2.31	10	rs9651453	106207209	C	*CCDC147*	−2.27
2	rs2345499	18336652	A		2.24	10	rs7089539	129816246	A	*PTPRE*	−2.21
2	rs295129	201229473	A	*SPATS2L*	−2.24	10	rs10829089	27126196	G	*ABI1*	−2.19
2	rs4233734	29637040	A	*ALK*	2.21	11	rs1551184	134085771	G	*NCAPD3*	−2.71
2	rs4907254	97549668	A	*FAM178B*	2.15	11	rs721607	56997832	A		−2.52
2	rs4952916	48769377	G	*STON1*	2.14	11	rs260815	103918740	G	*PDGFD*	−2.49
2	rs4384764	37590284	A	*QPCT*	2.10	11	rs4441010	11269086	A		2.09
2	rs17041095	51127789	A	*NRXN1*	−2.07	12	rs10784885	71387093	G		−3.10
2	rs1484465	80298707	A	*CTNNA2*	−2.06	12	rs10772689	13657684	G		−2.86
2	rs17416354	227770844	A	*RHBDD1*	2.02	12	rs4567507	92397369	G	*LOC256021*	2.72
3	rs1011609	87401121	A		−3.30	12	rs189339	66252294	A	*HMGA2*	−2.45
3	rs6437616	105505307	A	*CBLB*	−2.71	12	rs7976243	8086083	A	*SLC2A3*	−2.31
3	rs6777136	4952779	A		2.47	12	rs1144713	32261239	G	*BICD1*	−2.29
3	rs800086	144342672	A		−2.36	12	rs3819536	2436998	G	*CACNA1C*	−2.28
3	rs2292662	63897215	A	*ATXN7*	−2.25	12	rs7311068	74339372	G		−2.02
3	rs13077801	37870175	C		−2.25	12	rs10846720	125181917	G		−2.02
3	rs9829249	191862817	C	*FGF12*	2.08	13	rs3087339	96410115	A	*DNAJC3*	−2.73
3	rs1464065	124910439	C	*SLC12A8*	2.06	13	rs9571713	67693182	G	*PCDH9*	−2.66
3	rs702043	124911288	G	*SLC12A8*	−2.02	13	rs2057588	72465952	A		−2.10
3	rs11720157	2232424	A	*CNTN4*	−2.01	13	rs288726	107494419	A		−2.09
4	rs2608852	170014786	G		3.48	13	rs12865628	74316318	A	*KLF12*	2.04
4	rs4146484	73680605	A		−3.07	14	rs17103062	66336423	A		−3.46
4	rs10866333	170068750	A	*SH3RF1*	2.67	14	rs882731	95027844	G	*SERPINA4*	2.94
4	rs1473017	179323762	A		−2.60	14	rs741488	78643784	G		2.93
4	rs1519309	187015089	G		−2.48	14	rs8013121	43635179	C		−2.37
4	rs1995103	140466311	G	*SETD7*	−2.43	14	rs12433465	47473866	G	*MDGA2*	−2.16
4	rs4541571	66378192	G	*EPHA5*	−2.41	14	rs8020889	30077029	A	*MIR548AI*	−2.08
4	rs6814814	160884054	A		2.41	15	rs4476154	97933953	C		−2.79
4	rs1544392	95775101	G	*BMPR1B*	2.40	15	rs8030235	61843150	A		2.62
4	rs11945758	118667234	A		−2.33	15	rs667282	78863472	G	*CHRNA5*	−2.50
4	rs11941162	73784340	G		−2.31	15	rs7180035	25956104	A	*ATP10A*	2.42
4	rs4698756	110866442	G	*EGF*	2.25	15	rs782904	61366200	A	*RORA*	2.20
4	rs3775309	187513585	C	*FAT1*	−2.24	15	rs6495308	78907656	A	*CHRNA3*	2.19
4	rs1522075	17178058	A		−2.20	15	rs11639181	79024016	G		−2.11
4	rs11733843	95703047	A	*BMPR1B*	2.19	15	rs573922	58740094	G	*LIPC*	−2.09
4	rs1982121	162646894	A	*FSTL5*	−2.18	16	rs1651014	12737141	G		3.21
4	rs9307295	105405240	G	*CXXC4*	−2.18	16	rs9929593	6724106	A	*RBFOX1*	−2.55
4	rs13102386	57680805	G	*SPINK2*	2.08	16	rs3096299	89448663	G	*ANKRD11*	2.46
4	rs10005964	77470300	A	*SHROOM3*	−2.07	16	rs12927658	6701566	A	*RBFOX1*	−2.45
4	rs1893714	99510279	G	*TSPAN5*	−2.03	16	rs6497563	21966869	A	*UQCRC2*	−2.26
4	rs10019047	150459436	G		2.03	16	rs3096324	89422823	A	*ANKRD11*	−2.13
5	rs2400796	101775394	A	*SLC O 6A1*	−3.04	16	rs9927251	64164990	G		2.08
5	rs340419	178758909	A	*ADAMTS2*	2.54	17	rs8068425	78472301	A		−2.34
5	rs780401	87540210	A	*TMEM161B*	2.48	17	rs8079321	17760789	G	*TOM1L2*	2.08
5	rs6594961	115591333	A	*COMMD10*	−2.25	18	rs1550650	69782086	A		−2.03
5	rs1438946	152509001	G		−2.22	18	rs101941	44168443	A	*LOXHD1*	2.02
5	rs6888135	141254063	C	*PCDH1*	2.20	19	rs4805677	31809855	C	*TSHZ3*	3.47
5	rs4447967	79472714	G	*SERINC5*	−2.10	19	rs897786	13960386	G		2.26
5	rs12656374	16346572	G		2.10	19	rs395760	44214193	G		2.21
5	rs698912	74681773	G	*COL4A3BP*	2.01	19	rs4803027	39404800	A		2.13
6	rs6922743	63967017	G		−4.24	20	rs1006945	37458009	C	*PPP1R16B*	−2.96
6	rs2475787	86415236	G		−2.82	20	rs2315654	62399641	A	*ZBTB46*	−2.60
6	rs455726	111699368	G	*REV3L*	−2.70	20	rs6035688	20743417	G		2.21
6	rs6907250	11913942	A		−2.63	21	rs11700664	37642196	A	*DOPEY2*	−3.91
6	rs191833	5482597	A	*FARS2*	−2.55	21	rs1619656	25200439	A		−3.26
6	rs13219323	3712882	G	*BTNL2*	−2.41	21	rs3787732	37622498	A	*DOPEY2*	2.64

Chr, Chromosome; MA, minor allele.

### Association analysis

None of the analyzed demographic factors (age, gender, educational level) had a significant relationship with FC or GMV alterations in the SCZ group (data not shown). Regarding clinical factors, SCZ illness duration correlated with FC or GMV alterations while anti-psychotic agent dose exposure did not (data not shown). Surprisingly, gFCD values did not associate with FC or GMV alterations.

Compared to HCs, the SCZ patients had significant incidences of SNPs in *PDE4B, SATB2, SLC12A8, EGF, CACNA1C*, *MAG12*, *GABBR2*, *CACAN1C*, *PPP1R16B* (Figure 3). Only the SNP that was associated with the rs1006737 allele of *PDE4B* correlated significantly with GMV (*r* = 0:19; *p* = 0.015) and FC (*r* = 0.21, *p* = 0.0074) in SCZ patients after controlling for age, gender, education level, and group (Bonferroni corrected, Figure 2).

SNP components were transformed to Z scores and visualized at |Z| > 2. This threshold revealed 136 risk SNPs (see Manhattan plot of these data in Figure 2). Annotation of these 136 SNPs indicated that they were associated with 65 genes, notably including *PPP1R16B*, *GBBR2*, *PDE4B*, *CANCNA1C*, *SLC12AB*, *SATB2*, *MAG12*, and *SATB2.* One *PDE4B* SNP, namely that associated with the rs1006737 allele, correlated significantly with GMV (*r* = 0:19 *p* = 0.015) and FC (*r* = 0.21, *p* = 0.0074) in the SCZ patient group after controlling for age, gender, education level, and group (Bonferroni corrected, Figure 2). None of SNPs correlated with gFCD values.

GMVs with a high gFCD (rank order: left temporal lobe > right orbital frontal lobe > left hippocampus > left occipital lobe > right cerebellum) correlated with five MCCB dimension subscores (Figure 3). With respect to positive correlations, GMVs correlated most strongly with speed procession subscores (*r* = 0.69) and most weakly with social recognition subscores (*r* = 0.59). With respect to negative correlations, GMVs in the prefrontal lobe correlated negatively with visual learning subscores while GMVs in the anterior cingulate cortex correlated negatively with reasoning subscores, thus relating GMV-gFCD to cognitive impairments.

In summary, combining GWAS and fMRI techniques we found that *rs1006737* an SNP allele of *PDE4B*, associated with GMVs within the hippocampus, temporal lobe, amygdala, and cerebellum in patients with SCZ. Interestingly, our data also demonstrated that the presence of the SNP allele *rs1006737* was associated with decreased postcentral gyrus–amygdala FC and increased parietal lobe–cerebellum FC. Additionally, these FCs also correlated with altered GMVs and *rs1006737.*

## Discussion

In the present study we employed an integrated research strategy encompassing genotyping, cognitive function testing, and neuroimaging to investigate the impact of genetic variations on brain structure and function in patients with SCZ and thus produced evidence that may help to explain why cognitive impairments occur in this patient population. Overall, the present study supports the neurodevelopmental hypothesis of SCZ, which posits that stress events may alter the expression of predisposing SCZ-susceptibility genes and thereby lead to the pathogenesis of a schizophrenic episode (Vaskinn et al., 2014).

SZ is a highly heritable MPD in which a large number of SCZ-susceptibility genetic variations have been identified and characterized, especially since genome-wide association analysis has become feasible and affordable. We observed significant GMV reductions in the hippocampus, temporal lobe, amygdala, and cerebellum that were associated with SNPs in SZ-susceptibility genes including *GBBR2*, *SATB2*, *CACNA1C*, and *PDE4B*, which together with *EGF* have been identified as the top-contributing SNPs in SZ (Chen et al., 2019; Zhuo et al., 2019). Notably, these genes have been related to molecular pathways essential to cognitive functioning, including chemical synapses, cell junctions, and neuron projections and thus their alteration may contribute to the development of cognitive impairments in SCZ (Zhuo et al., 2020).

Our data demonstrating that SNPs in known SCZ-susceptibility genes, such as those listed above, can be associated with alterations in brain structure, as indexed with GMVs, and brain FC suggest that innate susceptibility genes may induce brain functional aberrations when the brain’s functional capacities cannot compensate for deficits, leading to structural alterations, such as GMV reduction (Berrettini, 2003). Moreover, our data demonstrating that SNPs that have been related to cognitive deficits were observed in our patients with SCZ support the view that SCZ-related cognitive deficits precede clinical schizophrenia onset (Green, 1996; Bowie and Harvey, 2006; Schaefer et al., 2013; Luo et al., 2018).

Importantly, the present data demonstrate three-way SNP- GMV-FC associations in patients with SCZ, thus providing clues regarding potential genetic bases of cognition impairments in SCZ. This work, together with prior studies (Jenkins, 2019), provides empirical evidence in support of a three-way SNP- GMV-FC interaction underlying cognitive impairment in SCZ. Given that impaired cognition is observed in other MPDs (McCleery and Nuechterlein, 2019), it would be of interest to examine how SNP- GMV-FC relationships in SCZ may compare to SNP- GMV-FC relationships in other MPDs, especially with respect to cognitive deficit severity, which has been shown to be worse in male schizophrenia patients, versus in female patients, and in patients with early onset of schizophrenia symptoms (McCleery and Nuechterlein, 2019).

SNP- GMV- FC relationships did not correlated with any dimensions of the MCCB is an unexpectedly. However, it is unclear why gFCD did not associate significantly with other brain indices in our study. If gFCD reflects connection number, then this lack of correlation may mean that SCZ patients may experience FC strength alterations without overall changes in connection quantity. This possibility remains to be examined in future research.

### Limitations

There are several limitations to our study. First, the patients all had chronic SCZ (illness duration >5 years). Over the course of a long-term mental, illness, cognitive impairments could be influenced by many factors. Hence, in a future study, first-episode patients and high-risk individuals (e.g., first-degree relatives of MPD patients) should be examined to elucidate the trajectory of cognitive impairment. Second, our patients were all being treated with high-dosage anti-psychotic agents, which themselves can affect cognitive performance. Third, although we used genetic techniques to test the relationship between SNPs and cognitive impairments, cognitive performance may be influenced by additional environmental factors. Fourth, our data do not explain the phenomenon of SNP- GM-FC bi-directional correlations with MCCB dimension sub-scores.

## Conclusion

The present findings support the hypotheses that SCZ-related cognitive impairments are related to genetic factors (H1) and brain structural and functional alterations (H2). Furthermore, these findings are consistent with the hypothesis that genetic factors and brain alterations may interact in causing cognitive impairments in the patients with SCZ (H3). The biological mechanisms by which genetic factor-related whole brain structural and functional alterations lead to SCZ-associated cognitive impairments remain to be elucidated.

## Data Availability

The original contributions presented in the study are included in the article/supplementary material, further inquiries can be directed to the corresponding authors.
